# Utilization and associated factors of traditional bone setting service among patients with musculoskeletal injuries in Northeast Ethiopia

**DOI:** 10.3389/fresc.2025.1484403

**Published:** 2025-04-08

**Authors:** Mengesha Alemu Seid, Betelhem Walelgn, Ashenafi Kibret Sendekie, Getachew Tesfaw Walle, Melkamu Adamu Geremew, Mikiyas Haylu Sahlu, Simachew Asress Mekonen, Biruk Beletew Abate

**Affiliations:** ^1^ College of Medicine and Health Sciences, Woldia University, Woldia, Ethiopia; ^2^ Department of Clinical Pharmacy, School of Pharmacy, College of Medicine and Health Sciences, University of Gondar, Gondar, Ethiopia; ^3^ Faculty of Health Sciences, School of Pharmacy, Curtin Medical School, Curtin University, Bentley, WA, Australia; ^4^ Department of Surgery, Woldia Comprehensive Specialized Hospital, Woldia, Ethiopia; ^5^ School of Population Health, Curtin University, Bentley, WA, Australia

**Keywords:** traditional bone setting, musculoskeletal injury, associated factors, Woldia, traditional medicine

## Abstract

**Background:**

Traditional bone setting is widely practiced in Ethiopia, despite the lack of standardized training and associated risks. This study aimed to assess the prevalence of traditional bone setting service utilization and associated factors among trauma patients at Woldia Comprehensive Specialized Hospital.

**Methods:**

An institution-based cross-sectional study was conducted between April 9 and May 18, 2024. A total of 420 participants were selected using a consecutive sampling technique. Binary and multiple logistic regressions were used to assess the association between the independent variables and traditional bone setting service utilization.

**Results:**

A total of 420 patients with orthopedic injuries participated in the study. The prevalence of traditional bone setting utilization was 55.2% (95% CI: 50.44, 59.95). In a multivariable regression model, rural residency (AOR = 1.56, 95% CI: 1.02, 2.39), low annual income (<21,000 Birr) (AOR = 4.06, 95% CI: 1.97, 8.37), use of health insurance (AOR = 0.63, 95% CI: 0.41, 0.95), and extremity trauma (AOR = 1.82, 95% CI: 1.11, 2.99) were significantly associated with traditional bone setting utilization.

**Conclusion:**

Traditional bone setting utilization is common among rural, poor, and uninsured patients. Further research may be important to ensure its appropriate utilization.

## Background

The World Health Organization (WHO) defines traditional medicine (TM) as the body of knowledge, skills, and practices based on theories, beliefs, and experiences indigenous to different cultures (1, 2). TM aims to maintain health and treat illness. Traditional bone setters (TBS) are untrained individuals who treat bone fractures and dislocations using methods passed down through generations (3). Widely accepted in Ethiopia, TBS is often preferred to conventional care due to factors such as cost, accessibility, and cultural beliefs (4, 5). The musculoskeletal system refers to the functioning of the locomotor system, which comprises intact muscles, bones, joints, and adjacent connective tissues. Musculoskeletal injury (MSI) includes over 150 different diseases and conditions that affect the musculoskeletal system. These are characterized by impairments in muscles, bones, joints, and adjacent connective tissues, leading to temporary or lifelong limitations in functioning and participation. Musculoskeletal conditions are typically characterized by persistent pain and limitations in mobility and dexterity, which reduce people's ability to work and participate in society. Pain in musculoskeletal structures is the most common form of non-cancer pain (6).

Musculoskeletal problems affect 1.3 billion globally, causing 121.3 thousand deaths and 138.7 million disability (7). In Africa, musculoskeletal injuries are prevalent (15%–93.6%), with increasing demand due to road accidents (8, 9). Fractures are a leading cause of disability, especially in low- and middle-income countries resulting in more than 10% of disabilities (10, 11). In sub-Saharan Africa, the demand for musculoskeletal treatment has increased as the number of road traffic accidents and musculoskeletal injuries has increased (12). Traditional medicine, used by 10%–40% globally (13, 14), is widely used for fractures in Africa (10). In Ghana and Kenya, 52%–78% and 84%, respectively, prefer traditional bone setters (10, 15).

Despite acknowledging the perceived benefits of TBS procedures in terms of accessibility, affordability, and perceived effectiveness, the community continues to grapple with significant concerns regarding their quality and outcomes (2, 16). However, TBS has been implicated in a substantial number of amputations resulting from gangrene (17, 18). The prevalence of complications following TBS treatment remains alarmingly high ranging from 56.91% to 58% (19, 20).

The escalating rate of accidents in Ethiopia, including those stemming from civil war and internal displacement, has further exacerbated the problem of fracture management. Malpractices in fracture treatment have led to a surge in complications, placing a heavy burden on the healthcare system and individual victims alike (21). The physical, economic, and social consequences of these complications extend far beyond the affected individuals and their families, ultimately impacting society. Our clinical observations have also revealed that simple fractures and subluxations can develop into life-threatening conditions following visits to TBS. Despite the detrimental effects of these practices, the underlying sociocultural, economic, and healthcare-related factors that influence their use remain poorly understood in our study setting. This lack of understanding hinders our ability to mitigate the debilitating complications associated with TBS.

To address this gap, it is essential to conduct comprehensive studies that better understand the factors associated with TBS utilization. Such research could inform strategies to reduce complications related to TBS utilization and promote a more integrated approach to healthcare. Therefore, this study aims to determine the prevalence of TBS utilization and identify the contributing factors.

## Methods

### Study design and setting

A hospital-based cross-sectional study was conducted from April 9 to May 18, 2024. The study was conducted at Woldia Comprehensive Specialized Hospital in the Amhara National Regional State, Ethiopia. Woldia Comprehensive Specialized Hospital is located 521 km North of Addis Ababa, the capital of Ethiopia, and 360 km from Bahir Dar, the capital of the Amhara region. The hospital serves over 2,500,000 people in Woldia town, North Wollo Zone, and neighboring regions (Tigray and Afar). It is the largest government hospital in North Wollo, serving approximately 170,000–200,000 people annually. The Orthopedic department treats approximately 5,000 patients annually, of which 2,500 have fractures (22, 23).

### Sample size and selection procedures

All patients with MSI who visited Woldia Comprehensive Specialized Hospital during the data collection period were included in the study sample. Patients who were mentally or critically ill and unable to communicate were excluded from the study sample. The sample size was determined using a single population proportion formula, considering a 95% confidence interval, a 46% proportion from a survey on traditional bone setting service users, and associated factors among people with trauma in the Mecha district (4). A 5% margin of error was also considered. After accounting for a 10% non-response rate, a sample size of 420 was determined using a consecutive sampling technique. Patient flow was assumed to be random, and double recruitment was prevented by using participants' registration numbers. Participants who met the inclusion criteria were sequentially included in the study as they arrived at the hospital, until the target sample size was reached. This approach was used because patient visits to the hospital are random.

### Study variables

#### Dependent variables

Traditional bone setting service utilization (yes = 1, no = 0).

#### Independent variables

##### Sociodemographic factors for patients

Age, sex, place of residence, religion, educational status, marital status, occupation.

##### Trauma-related factors

Type of trauma, sit of trauma, cause of trauma, time since trauma.

##### Healthcare-related factors

Reason for preference, types of intervention, cost of services, distance.

#### Operational definitions

Traditional bone setting practice: patients who visit TBS only and first TBS then hospital after having sustaining trauma.

#### Data collection tools and procedures

The data were collected using a structured questionnaire developed from the literature (3, 4, 16). The questionnaire was prepared in English, translated into Amharic, and returned to English for consistency.

The data were collected through face-to-face interviews using a structured questionnaire with three trained diploma nurses/midwives. One supervisor closely supervised the data collection process.

#### Data quality control and management

The data collectors received one day of training to ensure the quality of the data collection. Additionally, a pretest was conducted on 5% of the sample population from Mersa District Hospital to minimize information bias. After data collection, the data were checked daily for completeness and clarity. Supervisors conducted follow-ups and provided supervision throughout the data collection process.

### Data analysis

Data were entered into EpiInfo and then exported to SPSS for analysis. Descriptive analyses were conducted. Variables with a *P*-value of <0.2 from the binary logistic analysis were included in the final model. Multivariable logistic regression was then performed, and variables with *P*-values of <0.05 were considered significant factors. Results were presented using AOR and 95% CI. Model fit was assessed using the Hosmer–Lemeshow test, which showed a value of 0.87. Multicollinearity was checked using a correlation matrix, with values ranging from −2.78 to 3.46.

### Ethical considerations

This study was approved by the Institutional Review Board of Woldia University College of Health Sciences and Medicine. An official permission letter was issued to Woldia Comprehensive Specialized Hospital. The purpose of the study was explained to the selected participants. Verbal consent was obtained from participants aged >18 years and from parents for those aged <18 years. The collected information was kept confidential, and participants were informed of their right to withdraw from the study at any time during the interview.

## Results

### Sociodemographic characteristics of participants

A total of 420 participants were required, and all MSL-injured patients were involved in the study, resulting in a 100% response rate. The mean (±SD) age of the participants was 34.58 ± 15.15 years. More than half (52.4%) were in the age range of 19–39 years. The ages of participants ranged from 1 year to 89 years. Most study participants (58.36%) were rural residents, while the remainder were urban residents (Table 1).

**Table 1:** Sociodemographic characteristics of participants in Woldia Comprehensive Specialized Hospital, 2024.

Variables		Frequency	Percentage (%)
Sex	Female	198	47.1
	Male	222	52.9
Age group (years)	0–18 years	52	12.4
	19–39 year	220	52.4
	40–59 years	118	28.1
	60 and above years	30	7.1
	Mean age	34.58	15.15
Educational status	No formal schooling	137	32.6
	Primary and secondary	216	51.4
	College and above	67	16.0
Marital status	Married	211	50.2
	Single	147	35.0
	Divorced	31	7.4
	Widowed	31	7.4
Resident	Rural	245	58.3
	Urban	175	41.7
Annual income	Less than 21,000 birr	220	52.4
	21,000–30,000 birr	120	28.6
	30,000–36,500 birr	33	7.9
	Above 36,500 birr	47	11.2

### Injury-related characteristics

More than one-third (34.5%) of study participants suffered fractures, with 55.2% of them having closed fractures. Regarding the site of injury, 65.0% had injuries to their extremities, 26.7% to their shoulders, and 8.3% to their trunks. Regarding the cause of injury, 35.7% were caused by road traffic accidents, 31.9% by falls, 11.0% by congenital deformities (defects in bones, muscles, tendons, and ligaments present at birth, such as clubfoot, metatarsus abductus, muscular dystrophy, and polydactyly), and the remaining 21.4% by gunshots and violence, as shown in Table 2.

**Table 2:** Injury-related characteristics of participants in Woldia Comprehensive Specialized Hospital, 2024.

Variables		Frequency	Percentage (%)
Type of trauma	Fracture	145	34.5%
	Dislocation	105	25.0%
	Strain	85	20.2%
	Others	85	20.2%
Type of fracture (*n* = 145)	Open fracture	65	44.8%
	Closed fracture	80	55.2%
Site of injury	Extremity	273	65.0%
	Trunk	35	8.3%
	Shoulder	112	26.7%
Cause of injury	Fall down	134	31.9%
	Natural deformity (clubfoot)	46	11.0%
	Road traffic accident	150	35.7%
	Other	90	21.4%

### Healthcare facility-related characteristics

The majority of respondents (61.7%) treated at the hospital experienced significant improvement. However, nearly 6% of the participants treated at the hospital reported complications. Regarding treatment types, nearly one-third (31.2%) of participants underwent surgery. This was followed by the use of bandages (26.0%), medication (25.5%), and traction (17.4%) (see Table 3).

**Table 3:** Healthcare facility-related participants in Woldia Comprehensive Specialized Hospital, 2024.

Variables		Frequency	Percentage (%)
How many minutes taken to the hospital from your home?	≤30 min	29	6.9%
	30–60 min	116	27.6%
	60–120 min	188	44.8%
	>120 min	87	20.7%
What is the reason behind hospital is your choice?	Fear of malunion from TBS	47	11.2%
	Fear of stiffness	95	22.6%
	Amputation	58	13.8%
	Family/peer pressure	26	6.2%
	Good infection prevention	21	5.0%
	Prolonged healing at TBS	40	9.5%
	Better healthcare Providers’ competency	59	14.0%
	Better outcomes in hospital	74	17.6%
What type of treatment did you get in the hospital?	Medication	107	25.5%
	Surgery	131	31.2%
	Bandage	109	26.0%
	Traction	73	17.4%
What was the outcome of hospital treatment?	Improved	259	61.7%
	Cured	137	32.6%
	Complicated	24	5.7%
How much time do you frequently spend in the hospital?	<3 months	206	49.0%
	≥3 months	214	51.0%

### Prevalence of traditional bone setting service utilization

The prevalence of traditional bone setting service utilization was 55.2%, with a 95% confidence interval (CI) of 50.44–59.95. Massage was the most commonly used method of bone setting by traditional bone setters (33.2%). The second most commonly reported method was bamboo splinting (30.2%), followed by traction (23.7%) and traditional medicine (12.9%). The most frequently mentioned reason for visiting TBS was its low cost (cheap), followed by the rapid service (25.9%). Other reasons included peer pressure (16.8%), lack of awareness (15%), social acceptability (15%), and negligence (12%). Among all TBS users, 45.7% reported no improvement, while only 5.6% reported improvement from their injury.

### Factors associated with TBS utilization

In the bivariate analysis, factors such as residency, low annual income, use of health insurance, site of injury, frequency of hospital visits, time from home to hospital, and type of treatment were found to be statistically significant (*p* < 0.2). In the multivariable analysis, factors such as rural residence [AOR = 1.56; 95% CI (1.02, 2.39)], low annual income (<21,000 birr) [AOR = 4.06; 95% CI (1.97, 8.37)], annual income between 21,000 and 30,000 birr [AOR = 2.34; 95% CI (1.09, 4.98)], use of health insurance [AOR = 0.63; 95% CI (0.41, 0.95)], and trauma of extremities [AOR = 1.82; 95% CI (1.11, 2.99)] were statistically significant (Table 4).

**Table 4:** Factors associated with TBS utilization in Woldia Comprehensive Specialized Hospital, 2024.

Variables		Preference of TBS		COR (95% CI)	AOR (95% CI)	*p*-value
		Yes	No			
Residence	Rural	142	103	1.30 (0.88, 1.92)	1.56 (1.02, 2.39)*	0.033
	Urban	90	85	1	1	
Annual income in birr	<21,000	140	80	4.12 (2.08, 8.16)	4.06 (1.97, 8.37)**	0.000
	21,000–29,999	64	56	2.69 (1.31, 5.54)	2.34 (1.09, 4.98)*	0.035
	30,000–36,500	14	19	1.74 (0.68, 4.41)	1.49 (0.56, 3.99)	
	Above 36,500	14	33	1	1	
Use of health insurance	Yes	105	107	0.62 (0.42, 0.92)	0.63 (0.41, 0.95)*	0.031
	No	127	81	1	1	
Site of injury	Extremity	162	109	1.83 (1.18, 2.85)	1.82 (1.11, 2.99)*	0.013
	Shoulder	19	16	1.46 (.68, 3.14)	1.09 (0.47, 2.51)	
	Trunk	51	63	1	1	

*p-value <0.05; **p-value <0.01.

## Discussion

The findings of this study revealed that the prevalence of TBS utilization was 55.2% [95% CI (50.44, 59.95)]. This is consistent with a survey conducted in Wolaita Sodo, where the prevalence was 56.9% (20), highlighting that TBS is a significant treatment option for many individuals. However, this study's prevalence is higher than that found in other regions, such as Addis Ababa (29.9%), Tanzania (40%), Mecha district (46.45%), and North Central Nigeria (50%) (4, 13, 24, 25).

The higher uptake of TBS observed in this study may be attributed to socioeconomic and cultural factors specific to the study area. It is possible that individuals in this region have not received adequate health education or counseling on modern fracture management through health facilities. Additionally, the differences in TBS uptake could be related to variations in understanding and perceptions of TBS, as well as to political instability. For instance, in a study conducted in Addis Ababa, 58.9% of respondents were aware of the disadvantages of TBS, whereas 98.2% were aware of modern orthopedic treatments (24).

The utilization of traditional bone setting (TBS) in managing musculoskeletal injuries has been widely recognized in many regions, particularly in sub-Saharan Africa and parts of Asia, such as India (1, 6). While the uptake of TBS is prevalent among lower socioeconomic groups, the current study provides additional insight into the factors influencing its continued use, particularly in Ethiopia (2). Although TBS is commonly associated with the poor, as seen in other studies, our findings suggest that the geographic and socioeconomic context in which TBS is practiced plays a significant role in its utilization (3). Many studies have highlighted the cultural and historical significance of TBS in regions such as sub-Saharan Africa, where it is often the first line of treatment for musculoskeletal injuries, especially in rural areas with limited access to formal healthcare (4). In India, TBS has also been recognized as an integral part of the healthcare system, particularly in rural regions, despite the growing emphasis on modern medicine (5). Similar to these findings, our study found that a significant proportion of participants relied on TBS before seeking formal medical attention, highlighting the continued importance of traditional healing practices in managing injuries. One critical aspect of TBS utilization that this study contributes to is the socioeconomic and geographical factors that influence its uptake (2). While several studies, such as those by have discussed the impact of socioeconomic status on the use of TBS, our study underscores the need for targeted interventions to address misconceptions and ensure that TBS is integrated within the formal healthcare system where appropriate (7). Moreover, our findings suggest that TBS is often viewed with skepticism by healthcare professionals, a challenge similarly noted in other sub-Saharan African contexts (8). This study advocates for a greater recognition of TBS as a complementary form of care, encouraging collaboration between traditional healers and modern medical practitioners to improve overall healthcare delivery.

This study found a strong association between the choice of TBS and factors such as rural residence, low annual income, lack of health insurance, and extremity trauma. Specifically, participants from rural areas were twice as likely to use TBS compared to those from urban areas. These findings align with a study conducted in the Mecha district (4). This association may be attributed to higher health literacy among urban residents, who are more likely to be informed about modern healthcare options, including fracture management, than their rural counterparts. Additionally, limited access to healthcare services in rural areas may further contribute to the reliance on TBS.

Income was a significantly linked factor in this study. Patients with household annual incomes of <21,000 birr and between 21,000 and 30,000 birr were 4.06 times and 2.34 times more likely to use TBS, respectively, compared to those with annual incomes >36,500 birr. These findings are consistent with previous studies conducted in Ghana and Nigeria (16, 26). This relationship could be explained by the fact that traditional medicine is generally more affordable than modern medical treatments. Health insurance use was also an associated factor for TBS utilization. Participants with health insurance were 37% less likely to use TBS than those without insurance. This may be because individuals with health insurance have access to free treatment at healthcare centers, alleviating concerns about the cost of care.

Additionally, the site of injury was a significant factor. Patients with extremity injuries were 1.82 times more likely to use traditional bone-setting practices compared to individuals with trunk injuries. This finding is consistent with studies conducted in Mecha and rural areas of Nigeria (4, 5). The community likely perceives extremity injuries as less severe than trunk injuries and extremity injuries are seen as easier to manage through TBS (4).

The study's findings offer valuable insights into the role of TBS services in the management of musculoskeletal injuries. These findings complement existing practices and provide a foundation for educating and encouraging the public, particularly those who are skeptical about the safety and effectiveness of these services. Additionally, the information gathered should encourage health professionals, who may be indifferent or dismissive of traditional medicine, to recognize and appreciate traditional bone setting as a legitimate alternative healthcare practice within Ethiopia's broader healthcare system.

Future research should focus on the practices and appropriateness of traditional healers in the North Wollo Zone traditional bone setting area, as well as exploring TBS practitioners' perspectives on collaborating with modern medicine. The current study has strengths, such as using standard and validated tools for data collection based on primary data. However, it has limitations, including possible recall bias and the study's cross-sectional nature, which prevent the establishment of a causal relationship.

## Conclusion

This study found that the prevalence of traditional bone setting utilization is high. Rural residency, low annual income, lack of health insurance, and limb injuries contribute to the utilization of TBS. As TBS services are increasingly recognized in society, integrating them into the healthcare delivery system is recommended to prevent issues arising from TBS malpractices.

## Data Availability

The original contributions presented in the study are included in the article/Supplementary Material, further inquiries can be directed to the corresponding author.
